# Remedies: New and Old

**Published:** 1887-01-01

**Authors:** 


### REMEDIES: NEW AND OLD.

THERE are two ways of experimenting with new remedies. The first is to try them on yourself; the second, and more commonly employed method, is to give them to other people. The late Sir Robert Christison, who was a model physician and experimenter, generally began with himself. None of his old students are likely to forget the gusto with which he used to tell the story of his personal experiment with Calabar bean. This bean, as professional readers know, is a most potent drug. Some friend had sent Sir Robert a sample, and he was minded to try it upon himself one morning at the commencement of his daily shave. He had not proceeded far with this operation before he discovered that he had got more than he had bargained for, and he became overpoweringly and almost helplessly ill. He says, "I immediately swallowed the shaving water"—the readiest emetic at hand. Medical aid was sought, and after remaining in a critical condition for some hours, he completely recovered. One might have supposed that after such an experience neither he nor any other physician would be in haste to make a further personal experiment. But both he and many other enthusiasts have repeatedly made similar trials since the Calabar bean incident, only a little more preliminary care has perhaps been exercised. Some venerable dog, which had nearly had his day, has probably been privileged to have the very first taste of the new and possibly dangerous dose. When the experimenter can with safety try a remedy upon himself, there are obvious advantages in doing so. One result is that his experience is obtained at first hand,—a matter of no inconsiderable moment. This course can be easily pursued when the remedy is not one previously unknown to science, but merely a new combination of familiar drugs, or a new method of preparing them for use. Messrs. Burroughs, Wellcome, & Co. have sent to us a number of their preparations for experiment and trial. These include tablets, tabloids, and liquids of various substances, new and old. The tablets and tabloids are intended to be a kind of variation on pills—a most praiseworthy intention. Small and innocent as a "pill" appears, the name has become a classical synonym for everything that is nauseous and intolerable. To some people the sight or even the very mention of a "pill" is the signal for a gastro-intestinal protest which threatens to end in an immediate peristaltic revolution, or, as the vulgar would say, a "fit of vomiting." Now here is the opportunity of the modern and more elegant "tablet or tabloid." It has no history, no long-standing repute, and therefore, of course, no *bad* repute. It offers itself as a pleasing but effective substitute for an old and long-tried friend indeed, but a friend of a villainous reputation. There is an insinuating novelty about the tabloid which ensures for it a favourable trial. We have ventured, in the case of the "vegetable laxative," to follow the example of Sir Robert Christison, saving our canine friend any disagreeable experiences for the present. The great authority already quoted used to say that a perfect aperient should have three distinguishing characteristics. It should act quickly, safely, and pleasantly—*cito*, *tuto*, *jucunde*. We can affirm, with the most perfect sincerity, that the vegetable laxative tabloid fulfils these conditions excellently well. Let no one imagine that in choosing for experiment a laxative rather than the last new remedy for sea-sickness, or angina pectoris, we are wanting in scientific appreciation of the progress of therapeutics. We hold that a safe, pleasant, and effective aperient is a most valuable addition to the physician's armamentarium. Where a drug like urethane or strophanthus is used once the laxative will be employed a hundred times. The tablets and tabloids have a novel and pleasant appearance. We propose to return to these preparations again. In the meantime, we may say that if the others are equal in merit to the vegetable laxative they are worthy of the sincerest commendation.

